# Glymphatic System Impairment in Type II Diabetes Mellitus Adults

**DOI:** 10.21203/rs.3.rs-6467065/v1

**Published:** 2025-07-02

**Authors:** Bhaswati Roy, Veronica Lubera, Kamal R Singh, Anshita Singh, Megan Carrier, Sarah E Choi, Matthew J Freeby, Rajesh Kumar

**Affiliations:** University of California Los Angeles; University of California Los Angeles; University of California Los Angeles; University of California Los Angeles; University of California at Los Angeles; University of California Los Angeles; University of California at Los Angeles; University of California Los Angeles

**Keywords:** Sleep, Cognition, ALPS index, Epworth sleepiness scale, Magnetic resonance imaging, Diffusion tensor imaging

## Abstract

Type 2 diabetes mellitus (T2DM) is associated with multiple systemic complications, including cognitive decline and increased risk of neurodegenerative diseases. The glymphatic system, a brain waste clearance pathway, can be impaired from sleep disturbances common in T2DM, has not been examined. Therefore, the aim was to evaluate glymphatic system in T2DM subjects using diffusion tensor imaging along the perivascular space (DTI-ALPS) index. A total of 78 T2DM adults and 106 healthy controls underwent for brain MRI. Sleep issues were assessed using the Pittsburgh Sleep Quality Index (PSQI) and Epworth Sleepiness Scale (ESS), and cognition with the Montreal Cognitive Assessment (MoCA). Group differences in DTI-ALPS, sleep metrics, and MoCA scores were assessed with analysis of covariance (covariates, age, sex, and BMI). T2DM patients exhibited higher PSQI (p = 0.03) and ESS (p = 0.004), reflecting poorer sleep quality and increased daytime sleepiness. MoCA scores were significantly lower in T2DM adults (p = 0.001), with impairments emerged in visuospatial skills, attention, and language. Also, significantly reduced DTI-ALPS values appeared in T2DM over controls (p = 0.003). T2DM adults show impaired glymphatic function along with poor sleep quality and day-time issues. The findings indicate that glymphatic dysfunction potentially-driven by metabolic, vascular, and sleep-related disturbances may exacerbate cognitive deficits in T2DM adults.

## Introduction

Diabetes is a widespread chronic disorder, with a global prevalence of 10.5%; estimated 537 million adults (20–79 years), and expected to rise to 783 million by 2045 [1]. Type 2 diabetes mellitus (T2DM) accounts for more than 90% of total diabetes cases [2], and is associated with significant systemic complications, including cardiovascular, renal, and neural impairments. Along with brain tissue changes [3–5], T2DM increases the risk of cognition decline and neurodegenerative diseases, such as Alzheimer’s disease (AD) [6]. Multiple factors, including neuroinflammation, oxidative stress, and vascular changes in T2DM are recognized contributors to neural injury, yet the underlying mechanisms linking T2DM to progressive brain tissue changes are not fully understood. Recent studies suggest bidirectional links between T2DM and sleep disturbances, which play a critical role in cognitive health [7–9]. The glymphatic system, a recently identified waste clearance pathway in the brain is optimally regulated during sleep and may play a critical role in maintaining brain homeostasis. Dysfunction of this system could lead to neural changes in T2DM [10].

The glymphatic system, primarily active during sleep, facilitates the removal of neurotoxic waste products, including β-amyloid and tau protein, which are implicated in neurodegenerative diseases, including the AD [11]. The system relies on the coordinated function of aquaporin-4 (AQP4) water channels, situated in astrocytic end-feet near cerebral blood vessels, to facilitate the convective flow of cerebrospinal fluid and clear interstitial solutes from brain parenchyma [12]. Multiple animal studies have shown that glymphatic clearance can be compromised by factors, such as aging [13], high blood pressure [14], and metabolic dysfunction [15]. However, glymphatic system actions in humans with T2DM remains unexamined. Understanding the glymphatic status in T2DM adults could provide insights into the mechanisms of diabetes-associated cognitive decline and higher risks for AD and suggest potential therapeutic targets.

T2DM adults commonly experience various sleep issues, including poor sleep quality, insomnia, and increased daytime sleepiness, which can be worsen due to T2DM-related factors, such as obesity, inflammation, and insulin resistance. In addition, T2DM-related sleep disruptions may contribute to poor glycemic control, creating a bidirectional relationship [7]. Sleep impairment has been associated with onset of cognitive decline at a younger age, dementia, and increased risk of developing AD [16]. More specifically, a recent meta-analysis conducted between T2DM patients with and without sleep issues showed a 1.55, 1.65, and 3.78 increased risks of AD, cognitive decline, and preclinical AD, respectively [16]. The buildup of amyloid-plaques and hyperphosphorylation of tau proteins composing neurofibrillary tangles in AD leads to the improper functioning of neurons and their eventual death [17, 18]. Therefore, the glymphatic system has the potential to exacerbate risk of dementia and AD in T2DM patients and its mechanisms need to be studied.

This study aims to examine the functionality of the glymphatic system in T2DM individuals by utilizing advanced imaging techniques. Magnetic resonance imaging (MRI) based diffusion tensor imaging along the perivascular space (DTI-ALPS) index offers a non-invasive, valuable means to better understand the glymphatic system in T2DM adults. The DTI-ALPS index leverages the principles of DTI to quantify fluid flow dynamics along the perivascular pathways and has been used in multiple conditions [19, 20]. Therefore, the goal of our study was to examine glymphatic system function in T2DM adults in comparison to healthy controls using DTI-ALPS index.

## Materials and Methods

### Subjects

A total of 78 T2DM adults (39 male and 39 female) and 106 control subjects (53 male and 53 female) were recruited for this study. Demographic and clinical data are presented in Table 1. T2DM patient medication regimens were stable and each subject could lay supine for the MRI. Control subjects were healthy and had no history of hypertension or diabetes and were not on any medications that would lead to neural injury. T2DM patients were recruited from the UCLA Gonda Diabetes Center and healthy controls through flyer advertisement on the UCLA campus and the West Los Angeles area. Multiple conditions, including psychiatric disease (major depressive disorder, schizophrenia, and bipolar disorder), diagnosed neurological disorders (e.g., seizure history, traumatic brain injury), cardiovascular events, such as stroke or heart failure, structural chest or airway abnormalities affecting respiration, renal failure, dementia, cystic fibrosis, chronic obstructive pulmonary disease, substance dependencies, claustrophobia, body weight exceeding 160 kg due to MRI scanner limitations, or metallic implants were considered as exclusion criteria for both T2DM and controls. All T2DM and control subjects provided written informed consent before the study, and research protocol of this study was approved by the UCLA Institutional Review Board. In addition, all methods were performed in accordance with the relevant guidelines and regulations.

### Quality of Sleep and Day time Sleepiness

Both the T2DM and control groups completed questionnaires assessing sleep quality and daytime sleepiness levels. The Pittsburgh Sleep Quality Index (PSQI) was used to measure sleep quality, while daytime sleepiness was evaluated through the Epworth Sleepiness Scale (ESS). Both assessments are well-established tools for assessing sleep quality and fatigue. A score of 5–21 on PSQI indicates poor sleep quality and a score of 10 or higher on ESS suggests excessive day time sleepiness.

### Cognition Examination

Both T2DM and control subjects underwent the Montreal Cognitive Assessment (MoCA) test for rapid evaluation of multiple cognition sub domains, including visuospatial skills, executive functions, attention, memory, language, and orientation. A global MoCA score of 26 or more was considered normal [21].

### Magnetic Resonance Imaging

Brain imaging data were acquired using a 3.0-Tesla MRI scanner (Siemens Magnetom Prisma Fit, Erlangen, Germany), with participants positioned in a supine posture. To minimize head movement, foam padding was placed on either side of the head. High-resolution T1-weighted images were acquired with a magnetization-prepared rapid acquisition gradient-echo (MPRAGE) pulse sequence, with the following parameters: repetition time (TR) = 2200 ms, echo time (TE) = 2.41 ms, inversion time = 900 ms, flip angle = 9°, matrix size = 320 × 320, field of view (FOV) = 230 × 230 mm, slice thickness = 0.9 mm, and 192 slices. Proton density (PD) and T2-weighted images were obtained in the axial plane using a dual-echo turbo spin-echo sequence (TR = 10,000 ms; TE1,2 = 12, 124 ms; flip angle = 130°; matrix size = 256 × 256; FOV = 230 × 230; slice thickness = 3.5 mm). For DTI, data were collected using a single-shot echo planar imaging with a twice-refocused spin-echo pulse sequence (TR = 12,200 ms; TE = 87 ms; flip angle = 90°; bandwidth = 1,345 Hz/pixel; matrix size = 128 × 128; FOV = 230 × 230 mm; slice thickness = 1.7 mm, b = 0 and 800 s/mm^2^, diffusion directions = 30).

### Visual Assessment

High-resolution T1-, PD-, and T2-weighted images were visually assessed to identify any major brain abnormalities, including cysts, tumors, or significant brain infarcts. DTI images were also checked for artifacts related to imaging or head motion. No participants included in this study displayed major brain pathologies or imaging artifacts.

### DTI Indices and ALPS Measurement

**Figure 1:**
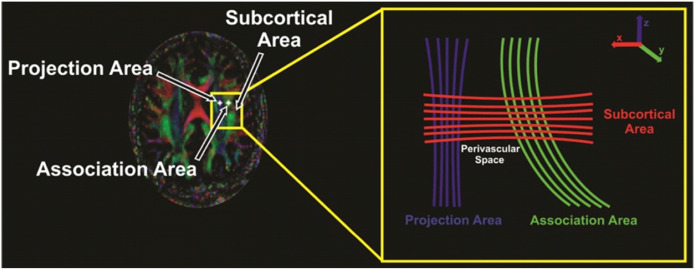
Regions of interest (ROIs) taken for imaging are marked with a white star and close-up panel shows different fibers running through the projection (blue), association (green), and subcortical (red) areas along with directionality.

Diffusion (b = 800 s/mm^2^) and non-diffusion (b = 0 s/mm^2^) weighted images were used to calculate diffusion tensor matrices, using the DTI-Studio software [22]. The average background noise level from outside the brain parenchyma was measured using diffusion- and non-diffusion weighted images to aid in removing of non-brain regions in the tensor calculation. Diffusivity maps, such as D_xx_, D_yy_, D_zz_, D_xy_, D_yz_, and D_xz_ maps were computed. The DTI-ALPS index was calculated following the methodology outlined in previous studies [20, 23]. Basically, the index was determined by analyzing diffusivity along the perivascular space’s direction over those of projection and association fibers on an axial slice at the level near the lateral ventricles, where the medullary veins are oriented perpendicular to the ventricular wall, which aligns with the x-axis, representing the perivascular space’s direction and the direction of both the projection (z-axis) and the association (y-axis) fibers is perpendicular to the direction of the perivascular space (Fig. 1).

All the diffusivity maps were normalized to Montreal Neurological Institute (MNI) space. Using unified segmentation method, non-diffusion weighted (b0) images were normalized to MNI space and these normalization parameters were applied to the diffusivity maps. Two sets of regions of interest (ROIs) were placed in the areas corresponding to the projection and association fibers at the level of the lateral ventricle body on the normalized diffusivity maps. These ROIs provided values for diffusivity parameters (D_xx_, D_yy_, D_zz_, D_xy_, D_yz_, and D_xz_) for each subject in projection and association fibers areas, and using these values, the ALPS index was calculated as: $\text{ALPS}\;\text{index}=\frac{\left(D_{\text{xxpro}}+D_{\text{xxasc}}\right)/2}{\left(D_{\text{yypro}}+D_{\text{zzasc}}\right)/2}$ where D_xxpro_ and D_yypro_ are D_xx_ and D_yy_ in the area of projection fibers and D_xxasc_ and D_zzasc_ are D_xx_ and D_zz_ in the association fiber areas.

### Statistical Analysis

Differences in demographics and clinical data were analyzed using independent samples and Chi-square tests using the statistical package for the social sciences (SPSS, v 29.0, New York, NY, United States). Diffusivity values and ALPS indices were compared between the T2DM and controls using analysis of covariance (SPSS Software; ANCOVA; covariates, age, sex, and BMI). The results were corrected for multiple comparisons using the Bonferroni correction methods. A value of p < 0.05 was chosen to establish statistical significance.

## Results

### Demographic and clinical variables

Demographic and other clinical variables of T2DM and control subjects are summarized in Table 1. No significant differences in age (p = 0.09) or sex (p = 1.0) observed between T2DM and control subjects. However, the body mass index (p < 0.001) was significantly higher in T2DM over controls. The ESS (p = 0.004) and PSQI (p = 0.03) scores were significantly increased in T2DM over control subjects. Global MoCA scores were significantly lower in T2DM compared to controls (p = 0.001), with significant differences in visuospatial (p < 0.001), attention (p = 0.001), and language (p = 0.009) sub domains.

### Diffusion and ALPS indices

**Figure 2:**
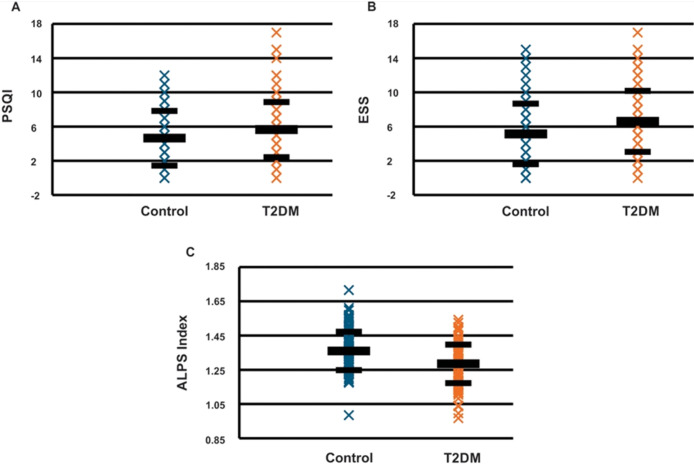
Scatter plots of perivascular space indexes in healthy controls and T2DM patients: (A) PSQI, (B) ESS, and (C) ALPS indexes.

**Figure 3:**
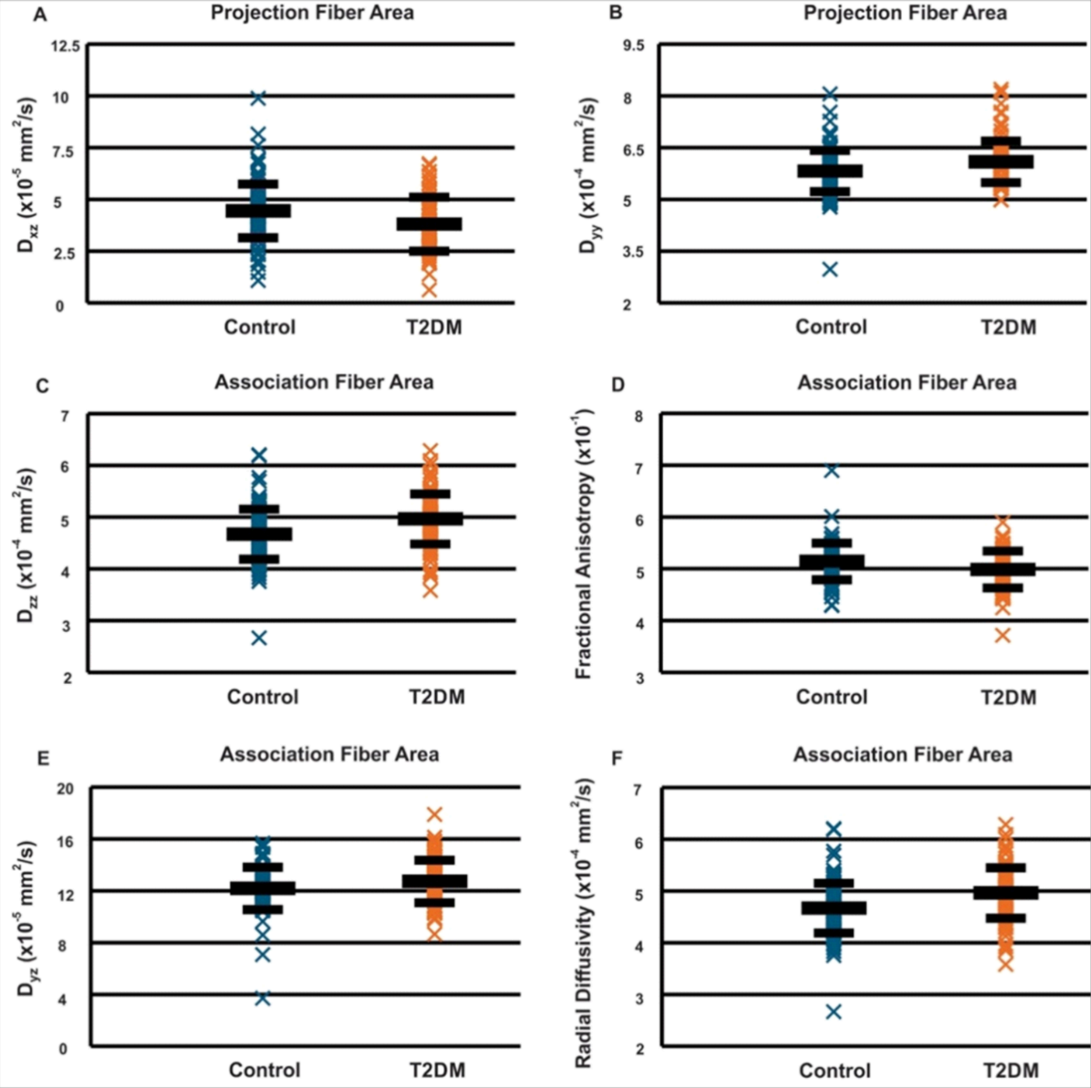
Scatter plots of perivascular space indexes in T2DM patients and healthy controls: (A) Dxz in projection fibers, (B) Dyy, (C) Dzz in association fibers, (D) fractional anisotropy, (E) Dyz, and (F) radial diffusivity.

The DTI-ALPS index (p = 0.003) was significantly decreased in T2DM compared to control subjects (Fig. 2). Also, D_xz_ and D_yy_ from projection fiber areas, and D_zz_ derived from association fiber areas (Fig. 3), were significantly different between T2DM and controls (Table 1).

## Discussion

We found significantly reduced DTI-ALPS indices, an indicator of impaired glymphatic system function, in T2DM over healthy controls. In addition, the diffusivity measures along projection and association fibers were altered in T2DM patients. The daytime sleepiness and sleep quality, measured using ESS and PSQI, showed enhanced daytime sleepiness symptom and poor sleep quality in T2DM over healthy controls. Also, the low MoCA scores observed in T2DM patients indicate early cognitive impairments associated with the condition. These findings suggest a critical interplay between metabolic dysregulation, glymphatic dysfunction, and sleep disturbances in T2DM adults that may contribute to the cognitive decline, as observed in this study, and pose early risks for dementia and AD.

The findings of this study provide evidence that glymphatic system function is significantly impaired in individuals with T2DM compared to healthy controls. The glymphatic system plays a key role in clearing metabolic waste and maintaining neural function and this impairment in glymphatic clearance may contribute to the increased vulnerability of the brain in T2DM adults, including tissue changes [3–5], to exacerbate neurodegenerative and cognitive disorders. In T2DM adults, metabolic dysregulation, inflammation, and vascular dysfunction are prevalent, and the compromised glymphatic function observed in our study could be an underlying factor linking the condition with increased neurocognitive risks.

Additionally, this study revealed that individuals with T2DM had higher ESS and worse PSQI scores, indicating greater daytime sleepiness and poorer sleep quality over healthy controls. Sleep is crucial for optimal glymphatic system function, as it is more active during sleep and facilitates cerebrospinal fluid flow, as well as enhances waste clearance from the brain parenchyma. The poor sleep quality observed in T2DM patients may further exacerbate glymphatic dysfunction, and this interplay creates a compounding cycle in which impaired sleep leads to reduced waste clearance, which in turn may contribute to cognition decline. These findings suggest that sleep disruptions in T2DM could be a driving factor in glymphatic system impairment and associated cognitive risks. Understanding this interrelationship highlights the importance of glymphatic system in T2DM and its potential as a therapeutic target to mitigate the early risks of Alzheimer’s disease and other neurodegenerative conditions in this high-risk patient population.

Recent animal studies investigating glymphatic system function in T2DM adults demonstrated alterations in the MRI markers of brain glymphatic measurements at both an early and advanced stage of diabetes suggesting a sensitive marker that could serve as an early diagnostic indicator for T2DM associated neurovascular damage and cognition decline [15, 24]. In addition, the dependency of glymphatic clearance and cerebrospinal fluid- interstitial fluid exchange on aquaporin-4 water channel in different neurological conditions is well known [13, 25–28], and the animal model of T2DM showed reducing trend in the aquaporin-4 expression with an increased disease severity of the condition, which indicate a similar mechanism may underlie glymphatic dysfunction in T2DM patients and suggest consideration of aquaporin-4 water channel is crucial for assessment of the glymphatic system status.

Previous studies on T2DM and metabolic syndrome have shown association with increased neuroinflammation, oxidative stress, and vascular abnormalities [29–31], and all of these can hinder glymphatic system functionality [32–34]. Such disruptions may interfere the clearance of beta-amyloids and tau protein, leading to their accumulation and an increased risk of neurodegeneration. Our study supports the evidence that metabolic and vascular dysfunctions in T2DM are likely contributors to the decline in glymphatic system efficiency. In our study, the glymphatic impairment and high ESS and PSQI scores suggests that metabolic disturbances in T2DM may not only affect peripheral organs, but may also disrupt key processes within the brain, further reinforcing the need for targeted interventions to address glymphatic function in T2DM adults.

Moreover, this study emphasizes the potential role of improving sleep in mitigating glymphatic dysfunction in T2DM patients. Given the established connection between glymphatic activity and sleep [10, 20], therapeutic approaches that improve sleep quality, such as cognitive-behavioral therapy, lifestyle changes, and possibly medications targeting sleep architecture, could support glymphatic function. These interventions could be particularly valuable for individuals with T2DM who exhibit poor sleep quality and high daytime sleepiness, as improving sleep could enhance waste clearance and reduce the neurocognitive burden associated with T2DM adults.

## Conclusions

This study provides robust evidence of impaired glymphatic function in individuals with T2DM adults, as reflected by significantly reduced DTI-ALPS index compared to healthy controls. Higher daytime sleepiness and worse sleep quality in T2DM patients further highlight the multifaceted impact of metabolic dysregulation impacting glymphatic system. The abnormal MoCA scores observed in T2DM patients emphasize the cognition impairments associated with glymphatic dysfunction and sleep disturbances. The observed glymphatic dysfunction, potentially driven by metabolic, vascular, and inflammatory abnormalities, suggests a mechanism linking T2DM with cognitive decline and the importance of targeting glymphatic system as a therapeutic strategy. Sleep-targeted therapies, such as cognitive-behavioral therapy, lifestyle modifications, and sleep-promoting medications, hold promise for mitigating glymphatic dysfunction and enhancing waste clearance in this high-risk population. The findings pave the way for research into therapeutic strategies that could improve glymphatic function and protect against neurodegenerative disorders and early risks for dementia and AD in T2DM adults.

## Figures and Tables

**Table 1:** Demographics, clinical variables, diffusivity and DTI-ALPS indices of T2DM patients and healthy controls.

Variables	T2DM (mean ± SD) [n=78]	Controls (mean ± SD) [n=106]	p values
Age (years)	56.5 ± 7.5	54.7 ± 6.5	0.09
Sex (Male:Female)	39:39	53:53	1.0
Ethnicity			
African American	7 (9.0%)	16 (13.2%)	
Asian	16 (20.5%)	35 (28.9%)	
Hispanic	24 (30.8%)	20 (16.5%)	
White	23 (29.5%)	44 (36.4%)	
Other	7 (9.0%)	6 (5.0%)	
Unknown	1	0	
BMI (kg/m^2^)	29.4 ± 5.0	26.3± 4.2	<0.001
Heart Rate (beats/min)	77.3 ± 11.8 (n=68)	71.7 ± 10.9	0.002
Systolic BP (mmHg)	127.8 ± 15.3 (n=69)	120.7 ± 17.2	0.006
Diastolic BP (mmHg)	78.6 ± 10.4 (n=69)	78.8 ± 14.2	0.92
PSQI	5.7 ± 3.6	4.6 ± 2.8 (n=105)	0.03
ESS	6.7 ± 3.9 (n=69)	5.1 ± 3.3	0.004
Diabetes Duration (years)	10.7 ± 8.1	-	
A1c	7.04 ± 1.3	5.3 ± 0.4 (n=52)	<0.001
MoCA Total	25.9 ± 2.5	27.1 ± 2.3	0.001
Visuospatial	4.2 ± 0.9	4.5 ± 0.7	<0.001
Naming	2.9 ± 0.3	3.0 ± 0.2	0.27
Attention	5.1 ± 1.1	5.6 ± 0.8	0.001
Language	2.2 ± 1.0	2.5 ± 0.7	0.009
Abstraction	1.9 ± 0.3	2.0 ± 0.2	0.13
Delayed Recall	3.5 ± 1.4	3.5 ± 1.5	0.98
Orientation	5.9 ± 0.2	6.0 ± 0.1	0.12

	Periventricular Projection Fiber Area (mean ± SD, × 10^−3^ mm^2^/s)			Periventricular Association Fiber Area (mean ± SD, × 10^−3^ mm^2^/s)		
	T2DM	Controls	p values	T2DM	Controls	p values
Dxx	0.72 ± 0.05	0.72 ± 0.05	1.0	0.69 ± 0.06	0.69 ± 0.06	0.43
Dxy	0.04 ± 0.01	0.05 ± 0.01	0.42	0.12 ± 0.03	0.12 ± 0.03	0.26
Dxz	0.04 ± 0.01	0.05 ± 0.01	0.001*	0.06 ± 0.01	0.06 ± 0.01	1
Dyy	0.61 ± 0.06	0.59 ± 0.06	0.03*	0.99 ± 0.06	1.01 ± 0.06	0.38
Dyz	0.20 ± 0.03	0.20 ± 0.03	0.26	0.08 ± 0.03	0.09 ± 0.03	0.03*
Dzz	0.93 ± 0.07	0.93 ± 0.07	1.0	0.50 ± 0.05	0.47 ± 0.05	<0.001*
	T2DM (mean ± SD)		Controls (mean ± SD)		p values	
ALPS	1.296 ± 0.11		1.35 ± 0.11		0.003	

ALPS = analysis along the perivascular space; T2DM = Type 2 Diabetes Miletus; SD = Standard Deviation; BP = Blood Pressure; BMI = Body Mass Index; MoCA = Montreal Cognitive Assessment; PSQI = Pittsburgh Sleep Quality Index; ESS = Epworth Sleepiness Scale, Dxx= diffusivity in x-direction; Dxy = diffusivity in x-y direction; Dxz = diffusivity in x-z direction; Dyy = diffusivity in y direction; Dyz = diffusivity in y-z direction; Dzz = diffusivity in z direction.

## Data Availability

The datasets generated during and/or analyzed in the current study are available from the corresponding author upon reasonable request.
